# Bis-indole-derived NR4A1 antagonists inhibit colon tumor and splenic growth and T-cell exhaustion

**DOI:** 10.1007/s00262-023-03530-3

**Published:** 2023-10-17

**Authors:** Kumaravel Mohankumar, Gus Wright, Subhashree Kumaravel, Rupesh Shrestha, Lei Zhang, Maen Abdelrahim, Robert S. Chapkin, Stephen Safe

**Affiliations:** 1 Department of Veterinary Physiology and Pharmacology, Texas A&M University, College Station, TX 77843 USA; 2 Department of Veterinary Pathobiology, Texas A&M University, College Station, TX 77843 USA; 3 Department of Medical Physiology, College of Medicine, Texas A&M University, College Station, TX 77843 USA; 4 Department of Biochemistry and Biophysics, Texas A&M University, College Station, TX 77843 USA; 5 Houston Methodist Cancer Center, Institute of Academic Medicine and Weill Cornell Medical College, Houston, TX 77030 USA; 6 Department of Nutrition, Texas A&M University, College Station, TX 77843 USA; 7 TAMU Flow Cytometry Facility, Texas A&M University, College Station, TX 77843 USA

**Keywords:** NR4A1, Antagonists, CRC, T-cell, Exhaustion, Inhibition

## Abstract

There is evidence that the orphan nuclear receptor 4A1 (NR4A1, Nur77) is overexpressed in exhausted CD8 + T cells and regulates PD-L1 in tumors. This study investigated the effects of potent bis-indole-derived NR4A1 antagonists on reversing T-cell exhaustion and downregulating PD-L1 in colon tumors/cells. NR4A1 antagonists inhibited colon tumor growth and downregulated expression of PD-L1 in mouse colon MC-38-derived tumors and cells. TILs from MC-38 cell-derived colon tumors and splenic lymphocytes exhibited high levels of the T-cell exhaustion markers including PD-1, 2B4, TIM3+ and TIGIT and similar results were observed in the spleen, and these were inhibited by NR4A1 antagonists. In addition, treatment with NR4A1 antagonists induced cytokine activation markers interferon *γ*, granzyme B and perforin mRNAs and decreased TOX, TOX2 and NFAT in TIL-derived CD8 + T cells. Thus, NR4A1 antagonists decrease NR4A1-dependent pro-oncogenic activity and PD-L1 expression in colon tumors and inhibit NR4A1-dependent T-cell exhaustion in TILs and spleen and represent a novel class of mechanism-based drugs that enhance immune surveillance in tumors.

**Supplementary Information:**

The online version contains supplementary material available at 10.1007/s00262-023-03530-3.

## Introduction

Colorectal cancer (CRC) is a highly complex disease with multiple risk factors including both genetic/heritable and environmental components that contribute to disease incidence [1, 2]. Despite enhanced participation in colon cancer screening and development of new treatment regimens there are over 1.8 million new cases and 900,000 deaths per year from CRC worldwide [3, 4]. Individuals with a family history of colon cancer or inherited genetic mutations have a high risk for of developing CRC,; however, it is estimated that 60–65% of all cases are “sporadic” with no inherited or genetic risk factors in their background [5]. In addition, there has been an increase in the incidence of CRC in young adults and the reasons for early onset of this disease are not well defined [6]. Colorectal cancer formation involves multistep initiation, promotion, progression and metastasis pathways and this is common for CRC and many other cancers. However, this multistep pathway develops differently for the adenoma–carcinoma (85–90%), serrated (10–15%) and inflammatory pathways where chromosomal instability (CIN) and the CpG island methylator phenotype (CIMP) are characteristic features of the former two major pathways, respectively [2].

Surgery is a major first treatment option for many colon cancer patients and this can also be accompanied or preceeded by radiation and neoadjuvant therapy. High-risk patients with stage III and IV colon cancer are treated with various cytotoxic drug combinations, and there is now increasing use of more targeted therapies in treating colon cancer, which are directed at specific genes and pathways [7–9]. Immunotherapies targeting checkpoint blockade represent a major advance in treatment of subsets of patients with melanoma and non-small cell lung cancer [10]. Clinical applications of checkpoint inhibitors for treating colon cancer are also effective for a subset of patients characterized “by a deficiency in mismatch repair (dMMR) resulting in high levels of microsatellite instability” [11]. Positive responses have been observed with antibodies targeting PD-1 (pembrolizumab and nivolumab), PD-L1 (durvalumab and atezolizumab) and CTLA-4 (ipilimumab), and the clinical applications of these checkpoint inhibitors in various combination therapies for treating colon cancer are ongoing [11–16].

The orphan nuclear receptor 4A1 (NR4A1, Nur77) is overexpressed in lung, colon, liver and breast cancers and in rhabdomyosarcoma and is a negative prognostic factor for lung, breast and colon cancer patient survival [17–25]. Ongoing research in our laboratories has been focused on the role of nuclear receptor 4A1 (NR4A1) in cancer and inflammatory diseases. NR4A1 regulates cancer cell proliferation, survival, cell cycle progression, migration, and invasion in multiple solid tumor-derived cell lines [18]. Bis-indole-derived (CDIM) compounds that bind NR4A1 act as NR4A1 antagonists and inhibit NR4A1-regulated pro-oncogenic pathways and genes in colon and other solid tumor-derived cells in culture and in vivo [17–23]. For example, the checkpoint gene PD-L1 is regulated by NR4A1 in breast cancer cells and treatment with CDIM/NR4A1 antagonists decreased PD-L1 and enhanced immune surveillance (increased CD8 + /CD4 + ratios) in syngeneic mouse breast cancer model [23]. Genome-wide studies have identified NR4A1 as a key mediator of T-cell dysfunction and NR4A1 also plays an important role in regulating genes which are involved in tumor-induced T-cell exhaustion [24–30]. PD-L1 and NR4A1 are also co-expressed in colon cancer cells and treatment with CDIM/NR4A1 antagonists also decreased PD-L1 levels in colon cancer cells [23]. Previous studies using mouse MC-38 cells show that in a syngeneic immunocompetent mouse model using this cell line as xenograft results in tumor growth and also splenomegaly and thus the effects of CDIM/NR4A1 antagonists in the spleen can also be investigated [32–35]. The advantages of this model in studying the effects of NR4A1 antagonists are also that modulation of T-cell exhaustion by these compounds can be observed in both tumor and spleen cell infiltrating lymphocytes. This study describes a novel approach for CRC therapy in which NR4A1 antagonists not only inhibit pro-oncogenic NR4A1-regulated genes/pathways but also enhance immune surveillance by targeting PD-L1 and reversing T-cell exhaustion in tumors and in spleen.

## Materials and methods

### Cells, growth, reagents and antibodies

Human colorectal cancer cells SW480, RK0 cells were purchased from ATCC, and murine colon cancer cells MC-38 were purchased from Kerafast. Cells were maintained in growth media and cell proliferation assays were determined as described using the MTT assay [20–25]. Antibodies, primers and oligonucleotides are summarized in Supplementary Table S1. 1,1-Bis(3΄-indolyl)-1-(3-bromo-5-trifluoromethoxyphenyl) methane (DIM-3-Br-5-OCF3) and 3,5-dichlorophenyl analog (DIM-3,5-Cl2) were synthesized by condensation of indole with 3-bromo-5-trifluoromethoxybenzaldehyde or 3,5-dichlorobenzaldehyde as described [17–22]. Indole and the substituted benzaldehydes were purchased from Sigma-Aldrich.

### Western blotting

SW480, RK0 and MC-38 cells were plated on 6-well plates at a density of 2X105 cells per well in DMEM supplemented with 2.5% charcoal-stripped FBS. After 24 h cells were treated with DMSO or different concentrations of DIM-3-Br-5-OCF3 or DIM-3,5-Cl2 for 24 h whole cell lysates were obtained with high-salt lysis buffer RRA (Thermo Scientific, Waltham, MA) and analyzed by western blotting as described [20–25].

### RNA interference

SW480, RK0 and MC-38 cells were seeded in six-well plates and allowed to grow to 60% confluence for 24 h. The cells were then transfected with 100 nmol/L of each siRNA duplex (see supplemental Table 1) for 6 h using Lipofectamine 2000 reagent (Invitrogen) following the manufacturer’s protocol, and 6 h after transfection, the medium was replaced with fresh medium containing 10% FBS and left for 72 h. Cells were then harvested and extracted, and protein expression was determined in western blots.

### Generation of NR4A1-deficient colorectal cancer cell line

Mouse NR4A1 CRISPR/CAS9 guide RNA gRNA1-CCGGGTAGCAGCCGTACACC (Exon-2), gRNA2- AAGCGCCAAGTACATCTGCC (Exon-3) and gRNA3-GTCCAAGTGTGCCCGGATGA (Exon-4) in a GenCRISPR eSpCas9-2A-GFP (PX458) vector was purchased from GenScript (Piscataway, NJ). MC-38 cells were then transfected with these plasmids. Three days after transfection, flow cytometry was used to collect high-GFP-expressing cells (single cells). These were seeded in 96-well plates (1 cell/well). After 2 to 3 weeks, cells from 72 individual wells were transferred and allowed to grow in 12-well plates. Proteins were extracted from these cells and screened by western blotting for NR4A1 expression. Best clones were selected, their genomic DNA was extracted, and PCR was performed with primers for three different exons. Purified PCR amplicons were sent for Sanger sequencing (Eton Bioscience, Inc., San Diego, CA). The sequences were analyzed for presence of insertion–deletions (INDELs) in the target region, and their clonal cells were grown into larger cultures. Also, protein expression of PD-L1 was followed in the two of the selected NR4A1 knockout clones by western blotting. A summary of the generation of the NR4A1- deleted cell line is given in Supplemental Fig. 1.

**Fig. 1:**
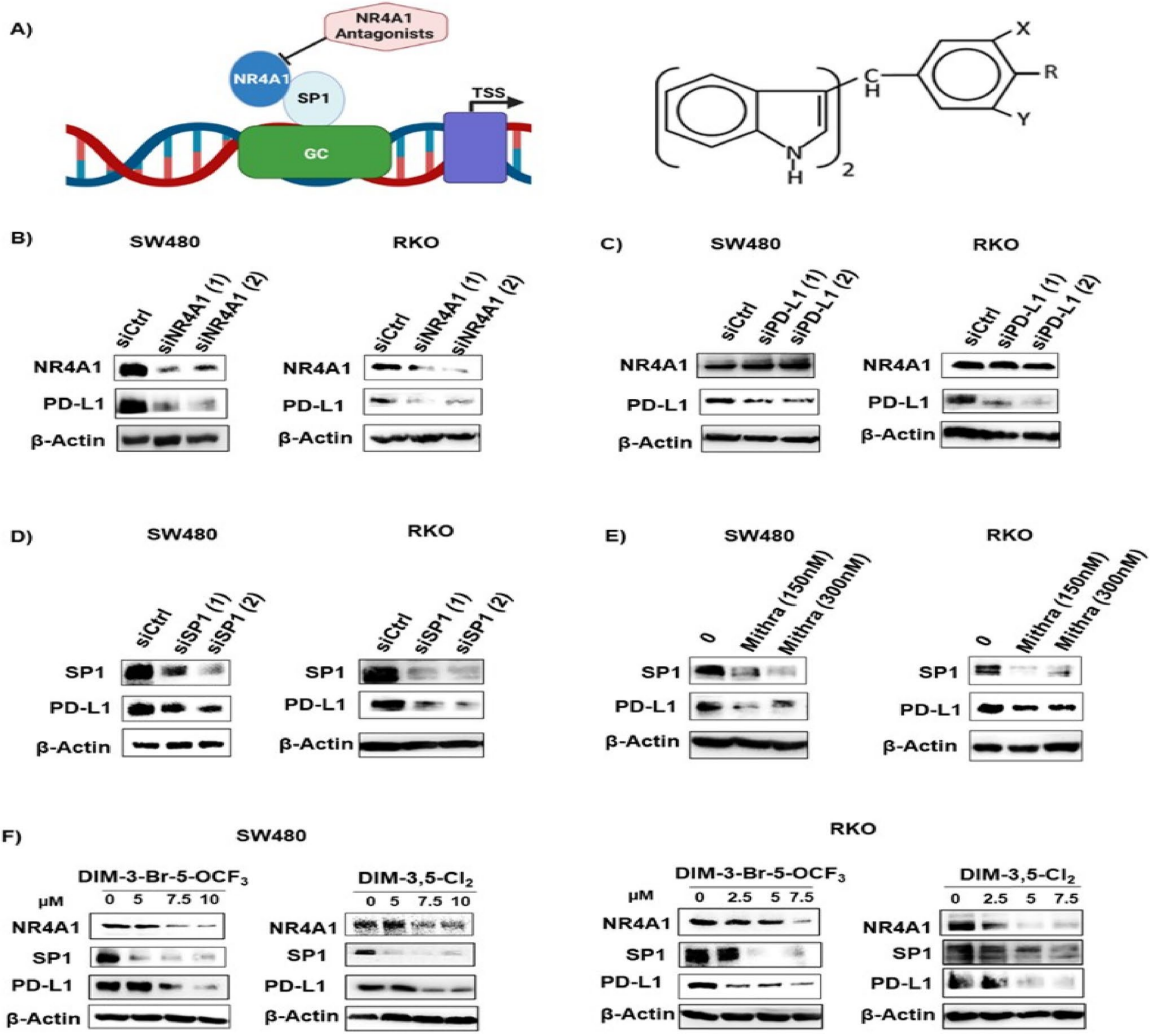
Regulation of PD-L1 by NR4A1/Sp1 in human colon cancer cells. **A** Model for regulation of PD-L1 and other genes with GC-rich promoters by NR4A1/Sp1 and structure of 3,5-disubstitutedphenyl DIM analogs. SW480 and RKO cells were transfected with oligonucleotides targeting NR4A1 **B**, PD-L1 **C** and Sp1 **D** or treated with mithramycin **E** and whole cell lysates were analyzed by western blots as outlined in Methods. SW480 and RKO cells **F** were treated with DIM-3-Br-5-OCF_3_ (X = Br, Y = OCF_3_, R = H) or DIM-3,5-Cl_2_ (X = Y = Cl; R = H) for 24 h and whole cell lysates were analyzed by western blot

### CD8 + T cell isolation and real-time PCR

CD8 + T cells were isolated from tumors using the Mojosort Mouse CD8 T Cell Isolation Kit (Biolegend, San Diego, CA) following the manufacturer’s guidelines. Total RNA was extracted from CD8 + T cells using Quick-RNA Miniprep Kit (Zymo Research, Irvine, CA) according to the manufacturer's instructions. The concentration and purity of the RNA samples were determined using a nanodrop spectrophotometer. Total RNA was reverse-transcribed, and real-time detection of specific mRNA was done with respective primers using iTaq Universal SYBR Green One-Step Kit (Thermo Fisher Scientific, Grand Island, NY, US) according to the manufacturer’s protocol with the CFX384 real- time polymerase chain reaction system (Bio-Rad). The comparative cycle threshold method was used for relative quantitation of samples. Values for each gene were normalized to expression levels of GAPDH. Primer sequences are summarized in Supplementary Table S1.

### Chromatin immunoprecipitation (ChIP) assay

SW480, RK0 and MC-38 cells (5X106) were plated and treated with DIM-3-Br-5-OCF3 or DIM-3,5-Cl2 for 3 h and subjected to ChIP analysis using the ChIP-IT Express magnetic chromatin immunoprecipitation kit (Active Motif, Carlsbad, CA) according to the manufacturer’s protocol. The primers used are given in Supplementary Table S1. PCR products were resolved on a 2% agarose gel in the presence of ethidium bromide.

### Syngeneic mice study

Female C57BL6 mice of 4–6 weeks old were purchased from Charles River Laboratories (Wilmington, MA). All the protocols for the animal studies were approved by the Institutional Animal Care and Use Committee (IACUC) at Texas A&M University. MC-38 cells (7.5 × 105 cells) were harvested in 100 μl of DMEM with ice-cold Matrigel (1:1 ratio). These cells were implanted subcutaneously into the right flank. After 2 weeks, mice were randomly divided into control and treatment groups of 6 animals each. Control group received 100 µL of vehicle (corn oil) Treatment groups received DIM-3-Br-5-OCF3–low dose (2.5 mg/kg/day) and high dose (7.5 mg/kg/day) or DIM-3,5-Cl2—low dose (2.5 mg/kg/day) and high dose (7.5 mg/kg/day) respectively in 100 µL volume of corn oil intraperitoneally for three weeks. All mice were weighed once a week over the course of treatment to monitor changes in body weight. Tumor volumes were measured using Vernier caliper over the treatment period. After three weeks of treatment, mice were killed, and body weights and tumor weights were determined. Spleen and tumors were collected for further processing.

### TIL and splenocytes profile analysis

Tumors and spleen were excised and disrupted mechanically with a surgical blade. For tumor samples, tumor digestion buffer was prepared in HBSS containing 400 U/mL collagenase IV (Worthington Biochemical Corporation, Lakewood, NJ) and 20 U/mL DNase I. Mechanically disrupted tumors were enzymatically digested using 500 µL of digestion buffer for every 200 mg of tumor. Spleen tissues were digested using mechanical disruption only. The cells suspensions were strained using 100 µm and then with 70 µm cell strainer and washed with 1XPBS and RBC lysis was carried out using ACK lysis buffer. The cell suspensions were again strained using 70-µm cell strainer and washed with 1XPBS. 100 µL of cell suspension was stained with 0.4% Trypan blue and counted. Cells were than resuspended in 1% PBS and analysis of immune cells was performed as follows:

Dead cells were labeled and eliminated from the analysis using Live/Dead Aqua Cell Stain Kit (Thermo Fisher, Carlsbad, CA) according to manufacturer’s protocol. A total of 4–6X106 cells/mL in PBSA were used for subsequent analysis. Fluorescent minus one (FMO) and compensation beads for all antibodies were prepared. All FMOs contain live and dead stain, whereas for compensation beads, just the antibodies with a drop of compensation beads were added following manufacturer's protocol. All samples were stained with Fc blocking solution (CD16/32) and stained with respective cell surface staining antibodies. Two separate panels were used for the flow cytometry experiments. The first panel (T-cell exhaustion panel) was composed of CD45 APC-Cy7, CD3 PE-Cy7, CD4 BV570, CD8a AF488, PD-1 PE-efluor 610, 2B4(CD244.2) BB700, LAG3(CD326) BV421, CTLA-4 APC, TIM 3 (CD366), BV711 and TIGIT PE. The second panel (Treg and transcription factor panel) was composed of CD45 APC-Cy7, CD3 PE-Cy7, CD4 BV570, CD8a PE-efluor 610, CD25 BB700, FOXP3 BV421 and Tbet BV605. For the second panel, samples were fixed and permeabilized using manufacturer protocol.

Stained samples were measured using the Luminex/Amnis Cell Stream flow cytometer. The BV421, Live/Dead Aqua, BV570, BV605 and BV711 were excited with the 405-nm laser at 100% power. The Alexa Fluor 488, PE, PE- eFluor 610, BB700, and PE-Cy7 were excited with the 488-nm laser at 100% power. The APC and APC-Cy7 were excited using a 640 nm laser at 100% laser power. The samples were run at a flow rate less than 1000 events per second. Spectral bleed through was compensated for using the Cell Stream auto compensation algorithm in the Cell Stream acquisition software. The flow cytometry data were analyzed using FlowJo software (Becton, Dickinson and Company). Gating strategies for both panels are depicted in Supplemental Figs. 2 and 3.

**Fig. 2:**
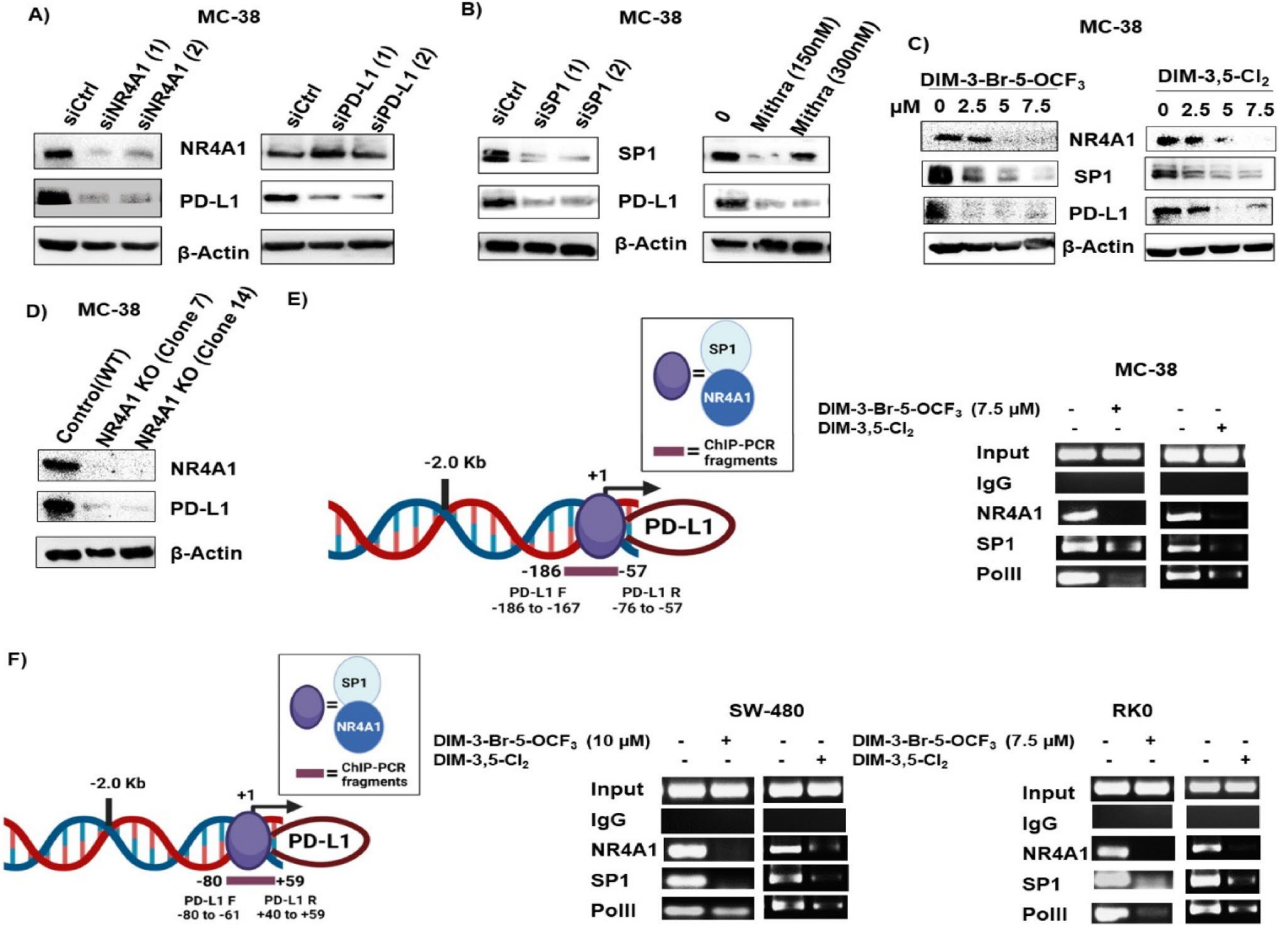
Expression of PD-L1 in mouse MC-38 colon cancer cells and regulation by NR4A1. MC-38 cells were transfected with oligonucleotides targeting NR4A1 and PD-L1 (**A**), Sp1 or treated with mithramycin (**B**) or treated with DIM-3-Br-5-OCF_3_ and DIM-3,5-Cl_2_ (**C**) or stable MC-38 NR4A1-KO cells generated by CRISPR/Cas9 and two clones (Clone 7 and Clone 14) (**D**), and whole cell lysates were analyzed by western blots. **E** Model of the GC-rich mouse PD-L1 promoter and primers targeting this region for the ChIP assay. MC-38 cells were treated with DMSO, 7.5 µM DIM-3-Br-5-OCF_3_ and 7.5 µM DIM-3,5-Cl_2_ and interaction with the GC-rich PD-L1 gene promoter was determined in a ChIP assay. **F** Model of the GC-rich human PD-L1 promoter and primers targeting this region in the ChIP assay; SW480 and RKO cells were treated as described and interactions with the PD-L1 promoter were determined in a ChIP assay as outlined in Methods. Figures were created partly with BioRender (https://app.biorender.com)

**Fig. 3:**
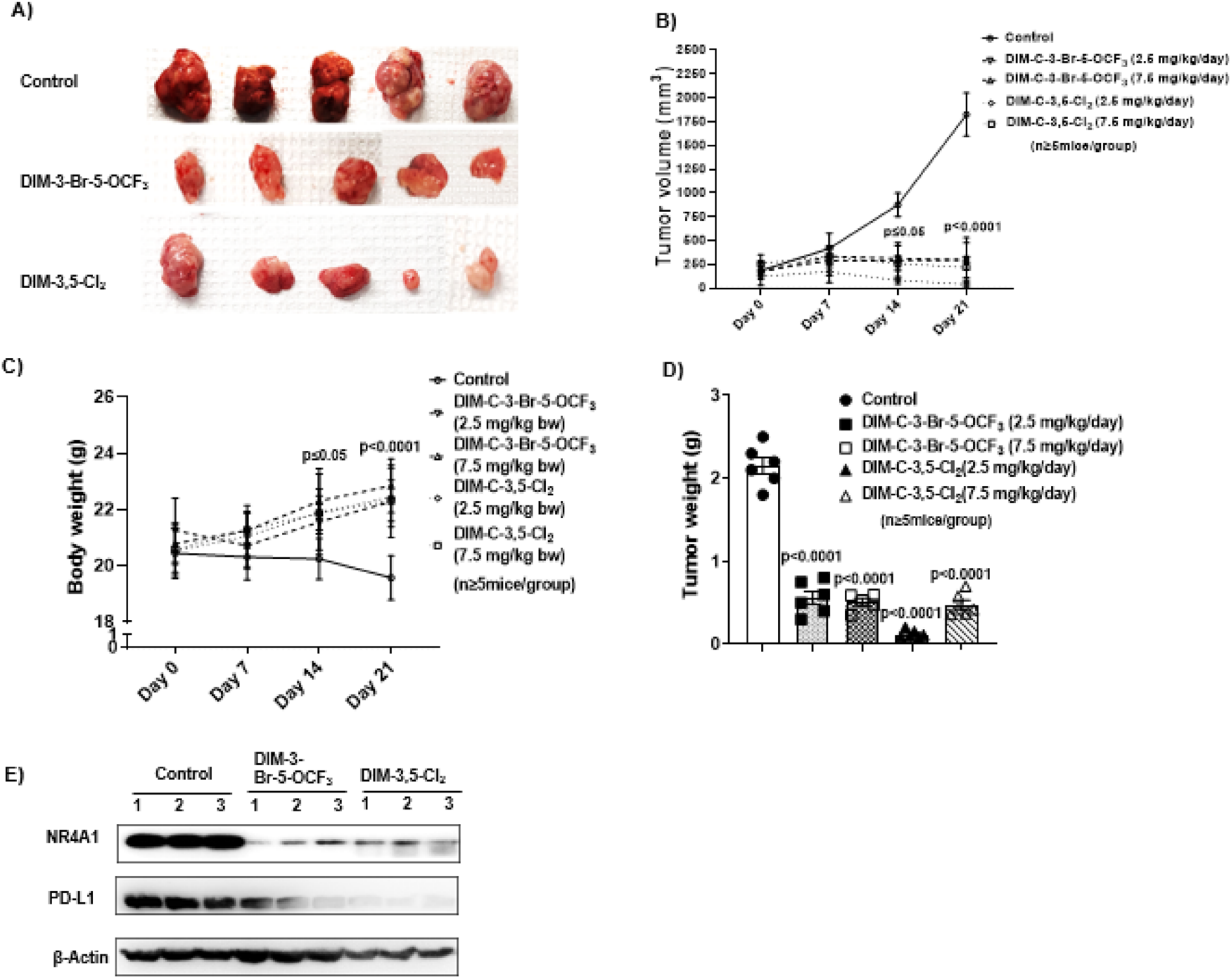
CDIM/NR4A1 antagonists inhibit colon tumor growth in a syngeneic mouse model. C57BL/6 mice bearing MC-38 cells as xenografts were treated for 21 days with corn oil (control), DIM-3-Br-5-OCF_3_ (2.5 and 7.5 mg/kg/d), DIM-3,5-Cl_2_ (2.5 and 7.5 mg/kg/d) and effects on tumor volume (**A**, **B**), body weight changes (**C**) and tumor weights (**D**) were determined as outlined in Methods. E. Tumor lysates from three animals per treatment group were analyzed by western blot as outlined in Methods. The Results are expressed as means ± SD. *n* = 6 mice/group. Significant (*p* values) induction or inhibition is indicated in graphs

### Statistical analysis

ANOVA and Fisher’s LSD were used to determine statistical significance. All experiments were repeated a minimum of three times. The data are expressed as the mean ± standard error (SE). *P* values ≤ 0.05 and lower were considered statistically significant. For the in vitro cell culture studies, an *n* of 3 was sufficient based on past experience and base on our prior and ongoing studies an *n* = 5–8 would provide a sufficient power (0.8) to detect significant differences between control and treatment groups in vivo.

## Results

### PD-L1 regulation by NR4A1

PD-L1 is regulated by NR4A1/Sp1 in breast cancer cells and treatment with CDIM/NR4A1 antagonists decreased PD-L1 protein levels and Fig. 1A illustrates the GC-rich PD-L1 promoter and the structure of the 1,1-bis(3’-indolyl)-1-(3,5-disubstitutedphenyl) methane (CDIM) compounds used in this study [23]. Figure 1B illustrates that PD-L1 levels are also high in RKO and SW480 colon cancer cells and knockdown of NR4A1 (Fig. 1B), PD-L1 (Fig. 1C), Sp1 (Fig. 1D) or treatment with mithramycin (Fig. 1E) or CDIM/NR4A1 antagonists (Fig. 1F) decreased PD-L1 levels these cells. Mithramycin is a specific inhibitor of Sp1 expression and binds to GC-rich promoters. The two CDIMs used were the most active inhibitors of colon cancer cell growth (Supplemental Fig. 4 and Supplemental Table S2). Knockdown of PD-L1 by RNA interference did not affect expression of PD-L1 (Fig. 1C).

**Fig. 4:**
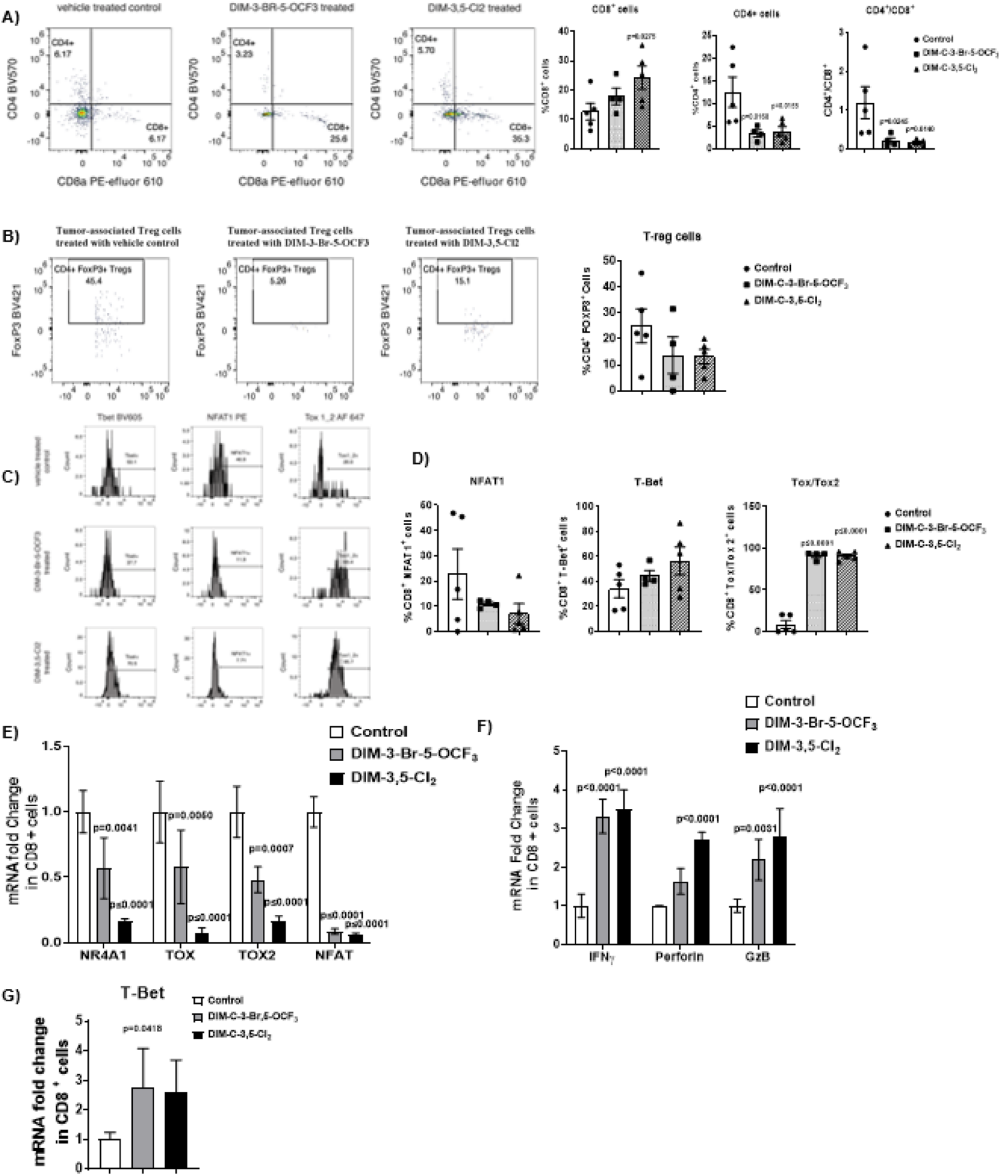
NR4A1 antagonists-induced changes in T-cell populations and transcription factors in CD8^+^ T-cells. Compound-induced changes on CD8^+^ and CD4^+^ T-cells (**A**) and Treg cells (**B**) in TILs was determined by flow cytometric analysis as outlined in Methods. **C** T-Bet, **D** NFAT1, and Tox/Tox2 expression in CD8^+^ T-cells was determined by flow cytometric analysis as outlined in Methods. Real-time PCR was used to determine mRNA levels of transcription factors (**E**), cytokines (**F**) and T-Bet (**G**) in CD8^+^ T-cells as outlined in Methods. Results are expressed as means ± SD. *n* = 5 for control and DIM-3,5-Cl_2_ groups, *n* = 4 mice in DIM-3-Br-5-OCF_3_ group. Significant (*p* values) induction or inhibition is indicated in graphs. The doses used were 2.5 mg/kg/d for both DIM-3-Br-5-OCF_3_ and DIM-3,5-Cl_2_

MC-38 mouse colon cancer cells also express NR4A1 and PD-L1 (Fig. 2A), and knockdown of NR4A1 by RNAi decreased expression of both PD-L1 and NR4A1; Sp1 knockdown or treatment with mithramycin also decreased Sp1 and PD-L1 expression in MC-38 cells (Fig. 2B) as observed in the human colon cancer cell lines, suggesting that PD-L1 may also be regulated by an NR4A1/Sp1 complex in MC-38 cells. Treatment of MC-38 cells with DIM-3,5-Cl2 and DIM-3-Br-OCF3 also decreased PD-L1, Sp1 and NR4A1 expression (Fig. 2C). In vitro NR4A1 gene disruption in MC-38 cells was carried out using CRISPR/Cas9 system (Supplementary Figure S1) expression of both NR4A1 and PD-L1 which were significantly reduced in NR4A1-KO MC-38 cells when compared to control cells (Fig. 2D). These data further support our initial finding that PD-L1 expression was regulated by NR4A1. Interactions of NR4A1 and Sp1 with the GC-rich proximal region of the human and mouse PD-L1 promoter were investigated in ChIP assays using primers that encompass the GC-rich sites of both promoters (Fig. 2E, F). In the untreated MC-38 cells (Fig. 2D) NR4A1, Sp1 and polII were associated with the GC-rich PD-L1 promoter and treatment with DIM-3,5-Cl_2_ or DIM-3-Br-OCF_3_ decreased these interactions. Similar results were observed in RKO and SW480 cells (Fig. 2F), indicating that PD-L1 is regulated by NR4A1/Sp1 in colon cancer cells as previously observed in human and mouse breast cancer cells [23] and NR4A1 antagonists decrease expression of this checkpoint gene product.

### In vivo tumor growth inhibition in a syngeneic mouse model

MC-38 cells as xenografts were used as an in vivo model to examine effects of CDIMs on PD-L1 and T-cells in TILs based on previous studies showing that this cell line was an excellent model for examining tumor-induced T-cell exhaustion markers [32, 33]. After tumors reached a size of approximately 100 mm3 animals were treated with both CDIM/NR4A1 antagonists at either 2.5 or 7.5 mg/kg/day for 21-days and compared to control (corn oil) animals, treatment with CDIMs significantly decreased tumor volumes (Fig. 3A, B). There were no apparent treatment-related toxicities induced by DIM-3,5-Cl_2_ or DIM-3-Br-5-OCF_3_ and compared to control mice those treated with CDIMs exhibited a slight increase in body weight (Fig. 3C) and both CDIMs also significantly decreased tumor weights (Fig. 3D). Western blot analysis of tumor lysates from three animals showed that both CDIM/NR4A1 antagonists (2.5 mg/kg/d) decreased expression of NR4A1 and PD-L1 (Fig. 3E).

### TILs: NR4A1 antagonists decrease T-cell exhaustion

Tumor-infiltrating lymphocytes (TILs) were isolated from control and NR4A1-treated mice (low-dose groups), and the percentage CD8^+^ and CD4^+^ T-cells and Treg cells were determined by flow cytometry. DIM-3,5-Cl_2_ but not DIM-3-Br-5-OCF_3_ significantly increased the percentage of CD8^+^ T-cells (Fig. 4A), whereas both compounds increased (not significant) the percentage of Treg cells (Fig. 4B). Flow cytometric of TILs also showed that DIM-3,5-Cl_2_ but not DIM-3-Br-5-OCF_3_ increased the percentage of T-Bet expressing cells and decreased the percentage of NFAT1-expressing cells (Fig. 4C) and this response is consistent with relief from T-cell exhaustion. However, the percentage of TOX1/2-expressing cells dramatically increased which was not expected. We also investigated CDIM/NR4A1 antagonists-mediated changes in expression of genes associated with T-cell exhaustion in CD8^+^ cells. Compared to the control cells DIM-3,5-Cl_2_ and DIM-3-Br-5-OCF_3_ decreased expression of NR4A1, and the high mobility group—box transcription factors TOX and TOX2, and NFAT in CD8^+^ T-cells (Fig. 4E). These results are also consistent with a role for NR4A1 antagonists in the reversal of T-cell exhaustion since NFAT, TOX and TOX2 in association with NR4A1 are important elements of CD8^+^ T-cell exhaustion [25]. CD8^+^ T-cell activation is also characterized by activation of cytokines, and Fig. 4F illustrates that TIL-derived CD8^+^ T-cells from mice treated with NR4A1 antagonists express higher levels of cytokine activation markers including interferon γ (IFNγ), granzyme B (GZB) and perforin mRNAs. In addition, we also observed that T-Bet mRNA (Fig. 4G) was induced by both compounds indicating that NR4A1 antagonists modulate expression of genes associated with the reversal of dysfunctional or exhausted T-cells. The TOX1/2 flow data and the mRNA expression data show opposite trends and this could be because of post-transcriptional and/or post-translational regulation of TOX1/2 in response to the CDIM inhibitor treatment.

**Fig. 5:**
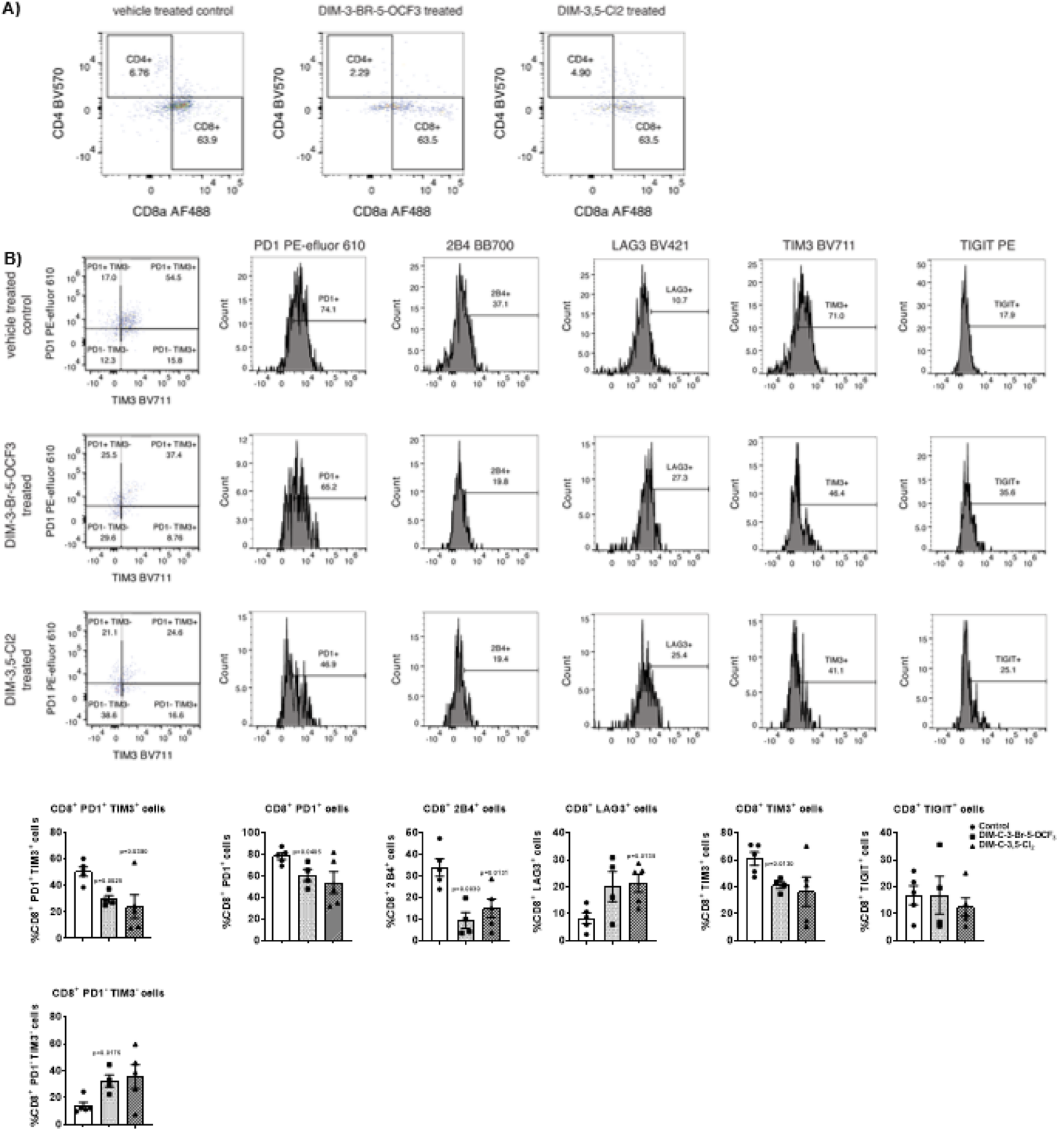
Flow cytometric analysis of CD8^+^ T-cells and surface markers associated with T-cell exhaustion in TILs from tumors. TILs were isolated from tumors derived from mice treated with corn oil (control), DIM-3-Br-5-OCF_3_ (2.5 mg/kg/d) and DIM-3,5-Cl_2_ (2.5 mg/kg/d). Flow cytometric analysis with specific antibodies were used to determine the percentage of CD4^+^ and CD8^+^ T TILs (**A**) and CD8^+^ TILs co-expressing PD1 and TIM3 and TILs expressing PD-1, 2B4, LAG3, TIM3, and TIGIT (**B**). The results are expressed as means ± SD. *n* = 5 for control and DIM-3,5-Cl_2_ groups, *n* = 4 mice in DIM-3-Br-5-OCF_3_ group. Significant (*p* values) induction or inhibition is indicated in graphs

TILs from control and treated mice were further investigated by flow cytometry to determine NR4A1 antagonist-induced changes in cell surface markers of T-cell exhaustion in the low-dose (2.5 mg/kg/d) treatment groups. The percentage of cells expressing the cell surface markers PD-1, 2B4, LAG3, TIGIT and TIM3 varied from approximately 7–75% in CD8^+^ T-cells derived from tumors of control (corn oil) animals, and this is consistent with T-cell exhaustion. However, after treatment with DIM-3,5-Cl_2_ or DIM-3-Br-5-OCF_3_ there was a decrease in the percentage of cells expressing these markers of T-cell exhaustion and this was most evident for PD-1, 2B4 and TIM3 in which significant decreases were observed (Fig. 5B). There was also an increase in the percentage of cells expressing LAG3 and TIGIT, however, these responses were not significant (Fig. 5B). We also observed a decrease in the percentage of cells co-expressing PD-1 and TIM3 (PD-1^+^ TIM3^+^) and an increase in the percentage of cells lacking co-expression of PD-1 and TIM3 (PD-1^−^ TIM3^−^) in response to the CDIM treatment. These data shows that the highly exhausted PD-1^+^ TIM3^+^ TILs are drastically decreased in the CDIM treatment groups.

**Fig. 6:**
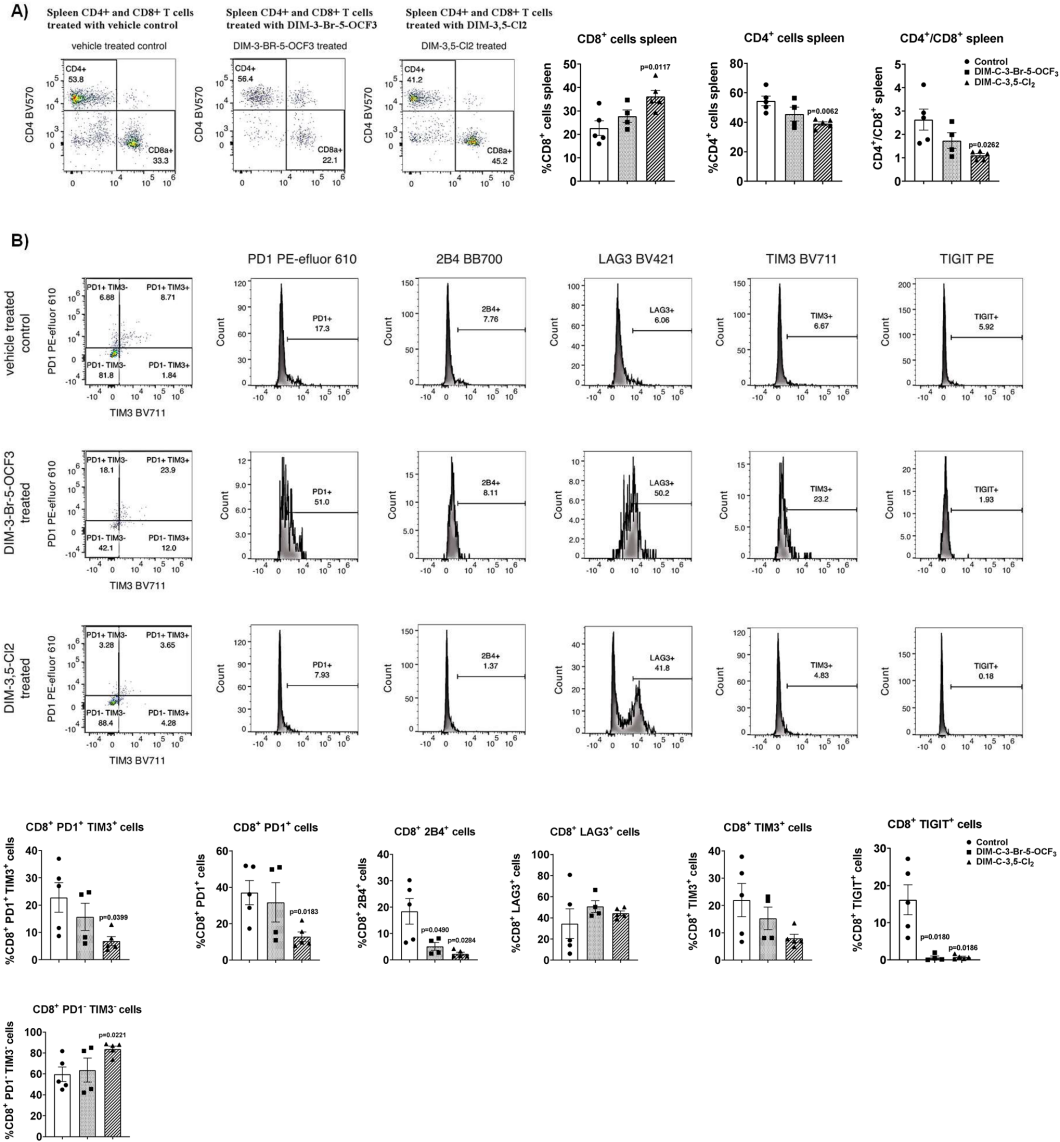
Analysis of splenic T-cells and markers of exhaustion in splenic CD8^+^ T-cells. Splenic lymphocytes were isolated and the percentage of CD8^+^ and CD4^+^ T-cells (**A**) were determined by flow cytometric analysis. Flow cytometric analysis using specific antibodies were used to determine the percentage of cells co-expressing PD1 and TIM3 and cells expressing PD-1, 2B4, LAG3, TIM3, and TIGIT (**B**). The results are expressed as means ± SD. *n* = 5 for control and DIM-3,5-Cl_2_ groups, *n* = 4 mice in DIM-3-Br-5-OCF_3_ group. Significant (*p* values) induction or inhibition is indicated in graphs. The doses used were 2.5 mg/kg/d for both DIM-3-Br-5-OCF_3_ and DIM-3,5-Cl_2_

**Fig. 7:**
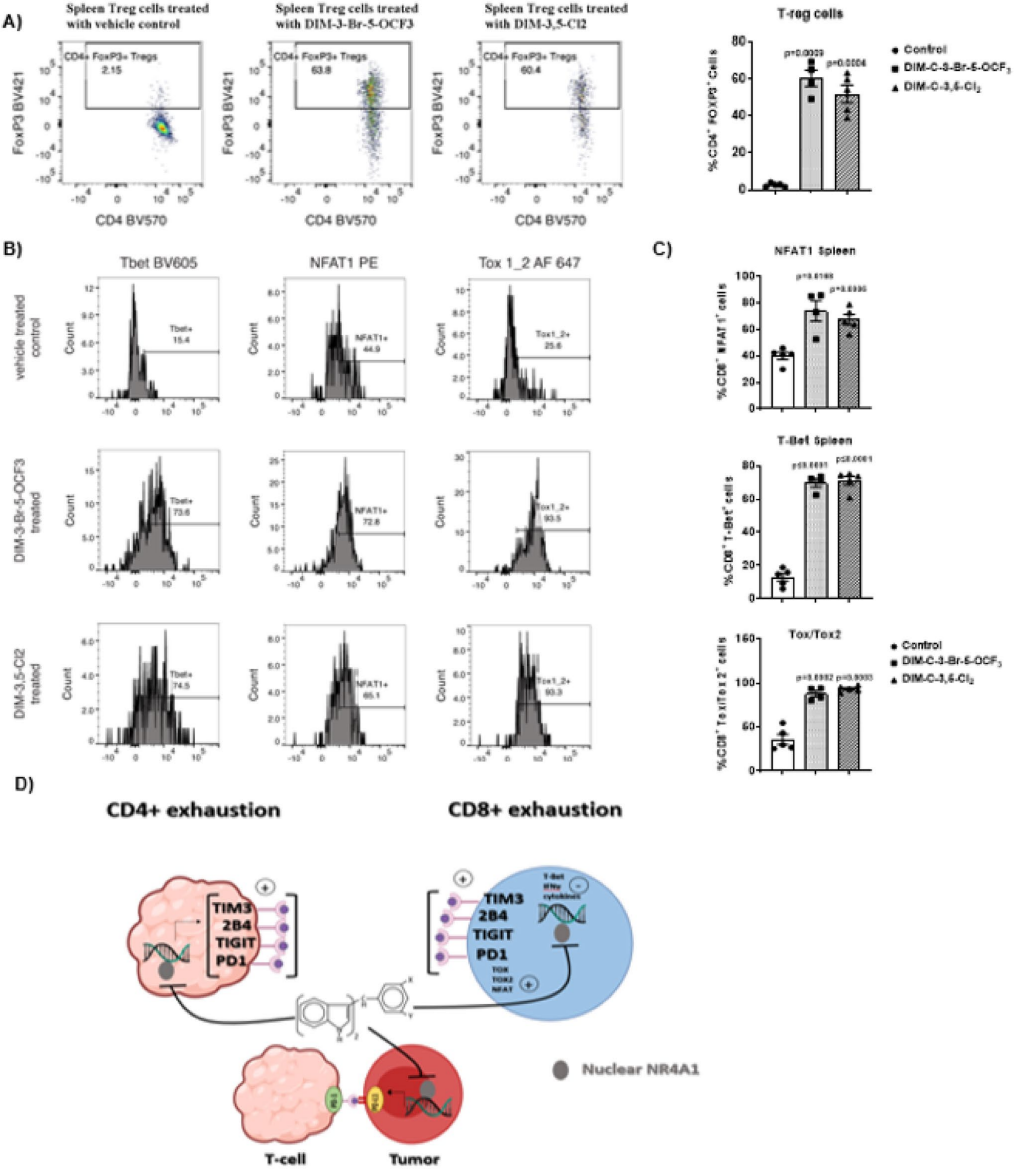
Flow cytometric analysis of cell surface markers associated with CD8^+^ T-cell exhaustion in spleen. Splenic lymphocytes were isolated from mice treated with corn oil (control), DIM-3-Br-5-OCF_3_ (2.5 mg/kg/d) and DIM-3,5-Cl_2_ (2.5 mg/kg/d) and flow cytometric analysis using specific antibodies was used to determine the percentage of regulatory Tcells (Tregs) (**A**) and CD8^+^ T-cells expressing transcription factors Tbet, NFAT1, and Tox1/2 (**B**, **C**). The results are expressed as means ± SD. *n* = 5 for control and DIM-3,5-Cl_2_ groups, *n* = 4 mice in DIM-3-Br-5-OCF_3_ group. Significant (*p* values) induction or inhibition is indicated in graphs. **D** Graphical summary of the effects of NR4A1 antagonists in tumors, spleen and lymphocytes (tumors and spleen)

Previous studies show that in immune competent mice bearing MC-38 cell-derived tumors there is also an increase in splenocytes and spleen weight [34, 35], and we observed that the NR4A1 antagonists decreased spleen weight (Supplemental Fig. 5B). The percentages of splenic CD8^+^ T-cells were increased in NR4A1 antagonist-treated mice relative to control-treated mice (Figs. 6 and 7A). The percentage of splenic CD8^+^ T-cells co-expressing exhaustion markers PD-1 and TIM3 and expressing the exhaustion markers PD1, 2B4, LAG3, TIM3 and TIGIT was 31.72, 45.18, 25.74, 45.34, 30.73 and 22.43% respectively (Fig. 6C–F, respectively) in control mice bearing MC-38—derived tumors, and these percentages were significantly decreased in mice treated with both NR4A1 antagonists. Thus, exhaustion markers in both TIL and splenic CD8^+^ T-cells were decreased by NR4A1 ligands.

Splenic regulatory Tcells (Tregs) were drastically increased in the spleen (Fig. 7B) in response to the NR4A1 inverse agonist treatment. CD8^+^ splenic Tcells also exhibited a higher percentage of Tbet-, NFAT1- and TOX1/2 expressing cells in the NR4A1 inverse agonist-treated cells relative to controls (Fig. 7C). The splenic CD8^+^ splenic T-cells show similar trends in the percentage of cells expressing Tbet and TOX1/2 transcription factors and the opposite trend for NFAT1 expression. The results demonstrate that NR4A1 plays an important intratumoral pro-oncogenic role in colon cancer and other solid tumors [18] and is also linked to T-cell exhaustion. Interestingly, these effects are attenuated by NR4A1 antagonists which target both tumors, spleen and T-cells (Fig. 7D) resulting in potent inhibition of colon tumorigenesis.

## Discussion

The function or lack of function of the immune system plays a central role in many diseases and the discovery and clinical applications of antibodies targeting checkpoints represent a major advance in cancer treatment [10, 36, 37]. Antibodies targeting the checkpoints PD-L1, PD-1 and CTLA-4 are currently being used or are in clinical trials for treating many cancers including CRC; however, there are a number of problems associated with treating cancer patients with immunotherapies. The percentage of patients that respond to immunotherapy is generally < 30%, and in many studies, some patients develop resistance and there are also toxic side-effects [40, 41]. Small molecular alternatives to immunotherapy are also being developed and show some promise. For example, small molecules such as metformin, the thalidomide-like drug pomalidomide, the JAK inhibitor SAR302503 cyclooxygenase inhibitors, and glycosylase inhibitors target PD-L1 and enhance immune surveillance [30, 40–43].

In contrast to the post-translational regulation of PD-L1 by glycosylase inhibitors [40], our previous study showed that NR4A1 regulates expression of PD-L1 in breast cancer cell lines and CDIM/NR4A1 antagonists decrease expression of this gene [23]. The results of studies in RKO, SW480 and MC-38 colon cancer cells show that knockdown of NR4A1 or treatment with DIM-3,5-Cl_2_ and DIM-3-Br-5-OCF_3_ decreases PD-L1 expression (Figs. 1, 2, 3). Previous studies in breast cancer cells show that NR4A1 acts as a cofactor of Sp1-mediated expression of PD-L1 [23], and our results confirm a similar pathway in colon cancer cells. The tumor growth inhibitory effects of CDIM/NR4A1 antagonists were observed in immune competent mice bearing MC-38 mouse colon cancer cells (Fig. 3) and this was accompanied by modest changes in CD8^+^ and CD4^+^ T-cells and Treg cells in TILs and a significant decrease in CD4^+^/CD8^+^ ratios (Fig. 4). We also observed that both NR4A1 antagonists inhibited tumor growth and downregulated PD-L1 in tumors demonstrating the highly effective intratumoral anticancer activity of these compounds.

The aggressiveness of colon tumors is not only associated with intracellular genes such as PD-L1 but also with increased expression of T-cell exhaustion markers in TILs from patients, which predict a poor prognosis [44, 45]. T-cell exhaustion is a complex immune cell deficit observed in viral and bacterial infections and in cancer where there is overexpression of multiple inhibitory receptors and impaired production of effector cytokines such as INFγ in CD8^+^ and CD4^+^ T-cells. Several studies using NR4A knockdown in mouse models have identified NR4A receptors and NR4A1 as important factors associated with T-cell exhaustion and the loss of NR4A enhances antitumor immunity [35–40].

Since NR4A1 is a key mediator of T-cell dysfunction [27] and has been associated with CD8^+^ T-cell exhaustion, we further investigated the potential role of NR4A1 antagonists DIM-3,5-Cl_2_ and DIM-3-Br-5-OCF_3_ in decreasing some of the markers of CD8^+^ and CD4^+^ T-cell exhaustion associated with the tumors and spleen. An immune competent syngeneic mouse model using mouse MC-38 colon cancer cells which express both NR4A1 and PD-L1 (Fig. 2) was used. Previous studies show that in the MC-38 cell-derived mouse tumor models there are also an increased splenic weight and evidence for T-cell exhaustion in spleen [34, 35, 45]. The precise functional role of NR4A1 in T-cell exhaustion has not been precisely defined but there is evidence for overexpression of this receptor and association with TOX and TOX2 transcription factors and also NFAT [25, 28]. The results illustrated in Fig. 4 show that in CD8^+^ T-cells isolated from TILs that treatment with NR4A1 antagonists decreased expression of NR4A1, TOX, TOX2 and NFAT. Moreover, these responses were accompanied by activation of T-Bet and cytokines which are typically observed during reversal or alleviation of T-cells from exhaustion. Thus, NR4A1 is linked to several markers of T-cell exhaustion that can be targeted by NR4A1 antagonists. Moreover, using several cell surface markers of T-cell exhaustion we also observed that NR4A1 antagonists decreased PD-1, 2B4, TIM3, and PD-1/TIM3 co-expression in both CD8^+^ T- cells isolated from tumors and spleen (Figs. 5, 6, 7; Supplemental Fig. 5). These results expand the role of NR4A1 in T-cell exhaustion and demonstrate the effectiveness of NR4A1 antagonists as anticarcinogenic agents is due to targeting the receptor in both tumors and T- cells (Fig. 7D). We acknowledge that the MC-38 cell line expresses the p15E retroviral antigen and it acts as a neoantigen in the TME and can increase the reactivity of the CD8^+^ TIL to the tumor cells. This can potentially confound the interpretation of the amount of CD8^+^ TILs in the tumor and their function. This could be why we see modest gains in CD8^+^ TILs in the tumor between treatments and vehicle. However, we think that the decrease in PD-1, 2B4 and TIM3 inhibitory receptors and NFAT1 transcription factor protein levels, TOX1/2 mRNA and NR4A1 mRNA levels along with a concomitant increase in Tbet protein expression and IFN-gamma, Granzyme B and perforin cytokine mRNA levels between the vehicle and CDIM-treated samples are increasing the antitumor function of the CD8^+^ TILs in the CDIM-treated samples. We hypothesize that in a different tumor cell line without the p15E antigen that the CD8^+^ TILs will be significantly more enriched in the CDIM-treated mice than the vehicle treated mice. Our future studies will investigate another tumor cell line that is known not to express p15E antigen.

In summary, results of this study show that NR4A1 antagonists inhibit colon tumor growth and decrease NR4A1-regulated expression of the checkpoint inhibitor PD-L1. These results are consistent with previous studies on colon and other solid tumor-derived cancers demonstrating the pro-oncogenic activity of NR4A1 and the inhibitory effects of NR4A1 antagonists [18, 22]. NR4A1 also plays a critical role in T-cell dysfunction, and this includes T-cell exhaustion [24–30], and our results using MC38 cells demonstrate the NR4A1 antagonists inhibit many of these dysfunctional NR4A1-dependent effects in T-cells and this includes reversal of several markers of T-cell exhaustion and activation of cytokines. The combined effects of NR4A1 antagonists in both tumors and T-cells (Fig. 7D) result in potent inhibition of colon tumorigenesis by targeting pathways/genes including PD-L1 in tumor cells and by enhancing immune surveillance through inhibition of NR4A1-dependent T-cell dysfunction in TILs and splenic infiltrating lymphocytes. However, it should be noted that these effects are observed in mouse models and in the future humanized animal models and the relative effects of NR4A1/ligands on tumor vs immune cells in their overall anticancer activities will be investigated. Future studies will also use CT26 mouse colon cancer cells and both orthotopic and genetic models of colon cancer to investigate the relative antitumorigenic effects of NR4A1 antagonists on tumors and immune cells and further develop optimal drug candidates for pre-IND and phase 1 clinical trials.

### Supplementary Information

Below is the link to the electronic supplementary material.

Supplementary file1 (DOCX 1183 kb)

## Data Availability

The data that support the findings of this study are available from the corresponding author upon reasonable request.

## Abbreviations

AMPK: AMP-activated protein kinase
ChIP: Chromatin immunoprecipitation
CIMP: CpG island methylator phenotype
CIN: Chromosomal instability
CDIM: Bis-indole derived
CRC: Colorectal cancer
DIM-3-Br-5-OCF3: 1,1-Bis(3΄-indolyl)-1-(3-bromo-5-trifluoromethoxyphenyl)methane
DIM-3,5-Cl2: 3,5-Dichlorophenyl analog
DMEM: Dulbecco's modified eagle medium
DMSO: Dimethyl sulfoxide
FBS: Fetal bovine serum
IACUC: Institutional animal care and use committee
IMDM: Iscove's modified dulbecco's media
NR4A1: Nuclear receptor 4A1
PBS: Phosphate-buffered saline
TILs: Tumor-infiltrating lymphocytes
