# Supplementary material for: Bis-indole-derived NR4A1 antagonists inhibit colon tumor and splenic growth and T-cell exhaustion
Source: Cancer Immunol Immunother. 2023 Oct 17;72(12):3985–99. doi: 10.1007/s00262-023-03530-3 (PMC10700478; doi:10.1007/s00262-023-03530-3)
Supplement: Supplementary file 1 — Supplementary file1 (DOCX 1183 kb) [file 262_2023_3530_MOESM1_ESM.docx]

**BIS-INDOLE DERIVED NR4A1 ANTAGONISTS INHIBIT COLON TUMOR AND SPLENIC GROWTH AND T-CELL EXHAUSTION**

Kumaravel Mohankumar^1^, Gus Wright^2,7#^, Subhashree Kumaravel^3#^, Rupesh Shrestha^4^, Lei Zhang^1^, Maen Abdelrahim^5^, Robert S. Chapkin^4,6^ and Stephen Safe^1*^

^1^ Department of Veterinary Physiology and Pharmacology, Texas A&M University, College Station, TX 77843 USA

^2^ Department of Veterinary Pathobiology, Texas A&M University, College Station, TX 77843 USA

^3^ Department of Medical Physiology, College of Medicine, Texas A&M University, College Station, TX 77843, USA

^4^ Department of Biochemistry and Biophysics, Texas A&M University, College Station, TX, USA, 77843

^5^ Institute of Academic Medicine and Weill Cornell Medical College, Houston Methodist Cancer Center, Houston, TX 77030, USA

^6^ Department of Nutrition, Texas A&M University, College Station, TX 77843, USA

^7^ TAMU Flow Cytometry Facility, Texas A&M University, College Station, TX 77843, USA.

^#^ Contributed equally to this work.

^*^ To whom correspondence should be addressed: Stephen Safe, Email: ssafe@cvm.tamu.edu; Tel. (979) 845-5988; Fax. (979) 862-4929

**Conflict of Interest:** The authors declare no conflict of interest.

**Supplemental Material**

Supplemental Figures S1-S5

Supplemental Table S1 and S2

**SUPPLEMENTAL FIGURES**


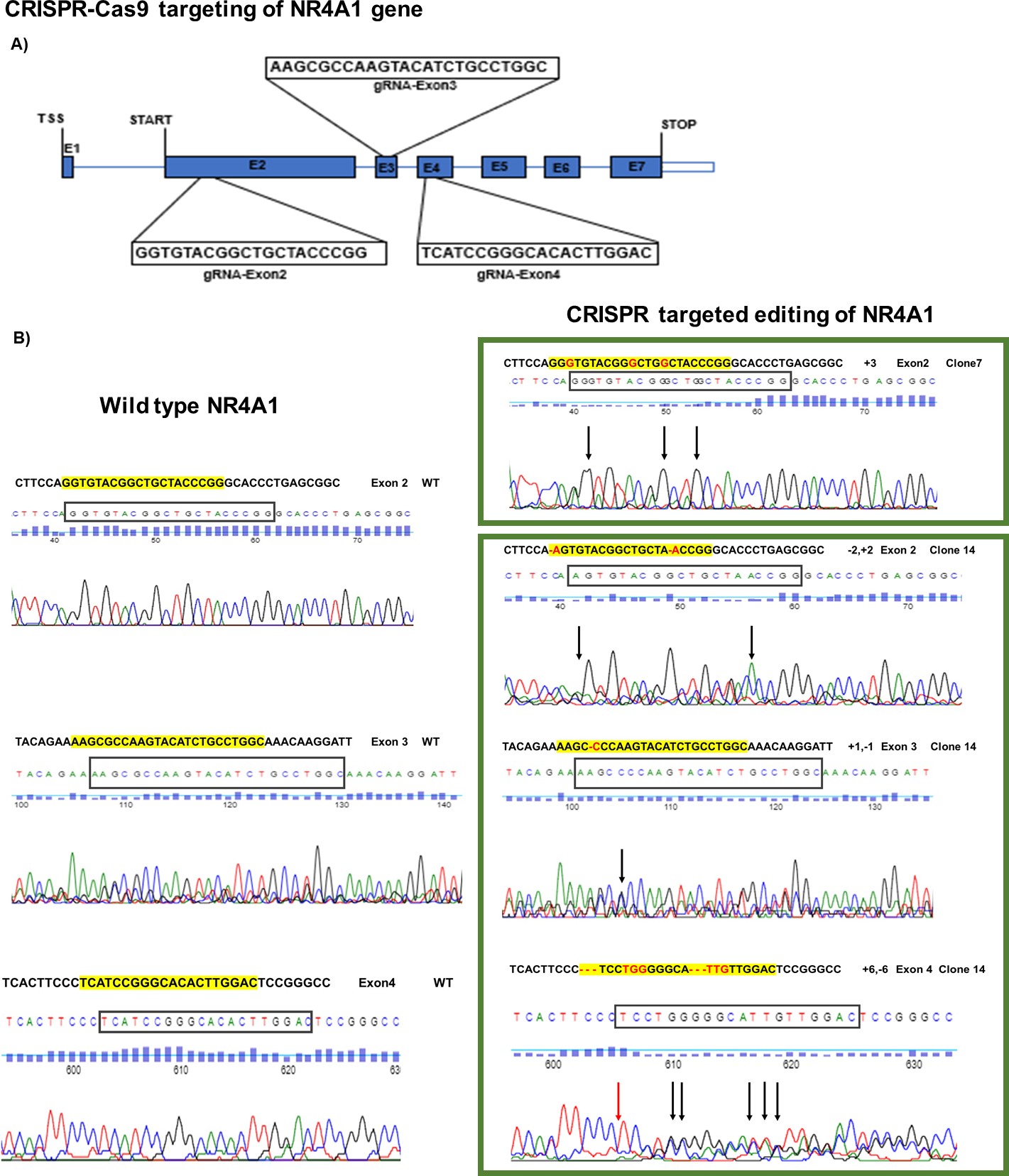


**Supplemental Figure S1.** CRISPR-Cas9 targeting of NR4A1 gene and sequencing electropherograms of PCR products. (A). Schematic diagram of sgRNAs chosen for different exons of NR4A1 gene. Three different sgRNA targeting sequences are highlighted in box. (B) Representative PCR amplicon sequences from wild type (control MC- 38 cells) and from 2 clones of CRISPR/CAS9 edited (with three different guide RNAs) NR4A1 exon 2, exon 3 and exon 4 were given.


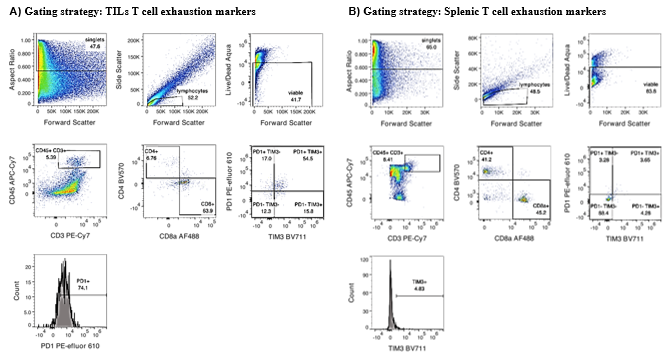


**Supplemental Figure S2.** Flow cytometry gating strategies for T cell exhaustion surface markers for TILs and splenic T cells. A.) Gating strategy for Tcell exhaustion surfaces markers in TILs. Working from the top left dot plot to the bottom left histogram. The top left dot plot depicts how the single cells were selected with the “singlet” gate. Lymphocytes were selected from the single cells using the “lymphocytes” gate in the forward scatter vs. side scatter dot plot. Viable cells were selected from the single, lymphocytes cells by using the “viable” gate depicted in the third dot plot. CD45 and CD3 expressing cells were selected from the single, viable cells using the “CD45^+^ CD3^+^” gate depicted in the fourth dot plot. CD4 and CD8a expressing cells were selected from the single, viable, CD45+ CD3+ cells using the “CD4^+^” gate and the “CD8a^+^” gate depicted in the fifth dot plot. The PD1^+^ TIM3^+^ expressing cells were selected from the single, viable, CD45^+^ CD3^+^, CD8^+^ cells using a quadrant gate “PD-1^+^ TIM3^+^”in the sixth dot plot. The first row of one-dimensional histograms is depicting the gating for PD-1^+^, expressing single, viable, CD45^+^, CD3^+^, CD8a^+^ cells. 2B4^+^, LAG3^+^ TIM3^+^ and TIGIT^+^ cells were gated similar to Figure 5 in the main text. B.) Gating strategies for the Tcell exhaustion surface markers in T cells in the spleen. The gating strategy description is similar to the TIL gating strategy described on the left.


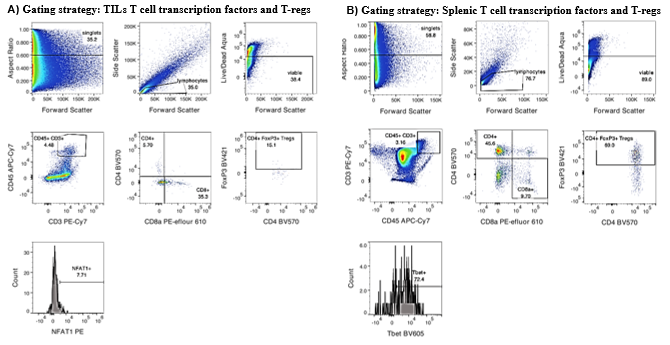


**Supplemental Figure S3.** Flow cytometry gating strategies for T cell transcription factors and regulatory T cells for TILs and splenic T cells. A.) Gating strategy for T cell transcription factors and regulatory T cells for TILs. Working from top left dot plot to the bottom left histogram. Single cells were selected using the “singlet” gate depicted in the top left dot plot. Lymphocytes were selected from single cells using the “lymphocytes” gate depicted in the forward scatter versus side scatter dot plot. Viable cells were selected from the single, lymphocyte cells using the “viable” gate depicted in the third dot plot. CD45 and CD3 expressing cells were selected from the single, lymphocyte, viable cells using the “CD45^+^ CD3^+^” gate shown in the fourth dot plot. CD4 and CD8a expressing cells were identified using the “CD4^+^” and “CD8a^+^” gates from the single, viable, CD45^+^, and CD3^+^ cells. Regulatory T cells were identified by gating on the FOXP3^+^ population from the single, viable, CD45^+^, CD3^+^, and CD4^+^ cells. NFAT1, TOX1/2, and Tbet expressing cells were selected with the “NFAT1^+^” and “Tbet^+^” gate from the single, viable, CD45^+^, CD3^+^, and CD8^+^ cells. TOX1/2 is gates are shown in Figures 4 and 7. B.) Gating strategy for T cell transcription factors and regulatory T cells for splenic T cells. The gating strategy description is similar to the TIL strategy on the left.


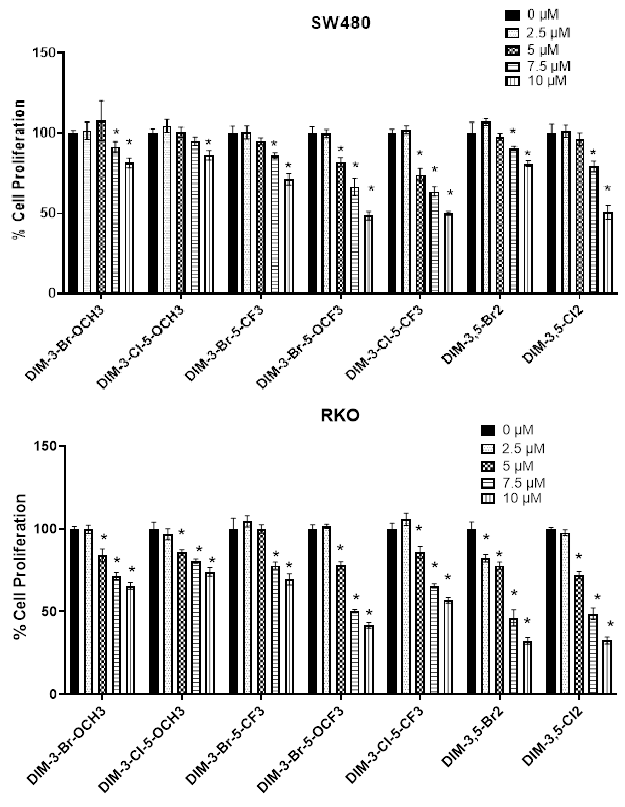

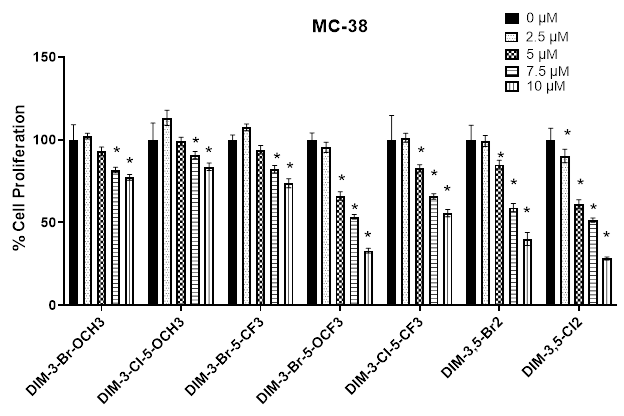


**Supplemental Figure S4.** Cell growth inhibition. SW480(A), RKO(B), and MC38(C) cells were treated with different concentrations of CDIM compounds for 24 hours and analyzed by the MTT assay as outlined in the methods. Results are expressed as means ± SD and significant(p<05) inhibition is indicated. IC50 values are given in Supplemental Table S2.


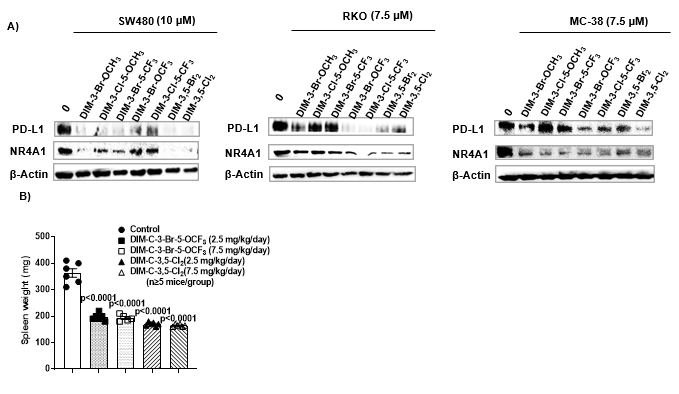


**Supplemental Figure S5.** Screening of 3,5-disubstitutedphenyl DIM analogs and effects of NR4A1 antagonists on spleen weight. A. Colon cancer cells were treated with DIM compounds for 24 h and whole cell lysates were analyzed by western blot. B. Spleen weights were determined after treatment of mice with corn oil (control) DIM-3-Br-5-OCF_3_ (2.5 mg/kg/d) and DIM-3,5-Cl_2_ (2.5 mg/kg/d). Significant (p<0.05) inhibition is indicated (*) and results are expressed as means ± SD for at least 4 mice per treatment group.

**SUPPLEMENTARY TABLES**

**Supplemental Table S1.** Summary of reagents, antibodies, immune staining sources, oligonucleotides and primers

| **Reagents** | **Source** | **Identifier** |
| --- | --- | --- |
| DMEM | Invitrogen | 11995-065 |
| Antibiotic | Invitrogen | 12450-062 |
| Fetal Bovine Serum (FBS) | Invitrogen | 26140-079 |
| Lipofectamine 2000 | Invitrogen | 11668-019 |
| Trypsin | Invitrogen | 25200-056 |
| Mithramycin | Cayman Chemical | 11434 |
| Chemiluminescence reagents | EMD Millipore | WBKLS0500 |
| PVDF membrane | Biorad | 1620184 |
| SDS Solution | National Diagnostic | EC-874 |
| Protogel (30%) | National Diagnostic | EC-890 |
| Protein Ladder | Biorad | 1610375 |
| Collagenase IV | Worthington Biochemical Corporation | LS004188 |
| DNase I | Zymo Research | E1010 |
| BD Perm/wash | BD Biosciences | 51-2091KZ |
| RBC Lysis buffer | eBioscience | 00-4333-57 |

| **Antibodies** | **Source** | **Identifier** |
| --- | --- | --- |
| PD-L1 (Human Specific) | Cell Signaling Technology | 13684s |
| PD-L1 (Mouse Specific) | R&D System | Mab90781 |
| Secondary anti-Rabbit-HRP | Cell Signaling Technology | 7074s |
| Secondary anti-Mouse-HRP | Cell Signaling Technology | 7076s |
| NR4A1 | Abcam | ab109180 |
| Sp1 | Abcam | ab13370 |
| Sp1 | Santacruz Biotechnology | sc-17824X |
| Β-actin | Sigma Aldrich | A5316 |
| RNA Pol II | Active motif | 102660 |
| Anti-mouse CD45 | BD Biosciences | 557659 |
| Anti-mouse CD3 | BD Biosciences | 560591 |
| Anti-mouse CD4 | BioLegend | 100541 |
| Anti-mouse CD8 | BD Biosciences | 557688 |
| Anti-mouse CD244.1 (2B4) | BD Biosciences | 745953 |
| Anti-mouse TIGIT | BD Biosciences | 565168 |
| Anti-mouse CD366 (TIM3) | BD Biosciences | 747622 |
| Anti-mouse CD279 (PD-1) | Invitrogen | 61-9985-82 |
| Anti-mouse T-bet | BioLegend | 644817 |
| Anti-mouse CD16/32 (Fc blocker) | Invitrogen | 14-0161-82 |
| Anti-mouse Foxp3 | BD Biosciences | 562996 |
| Anti-mouse CD25 | BioLegend | 102049 |

| **Resources (Assay kit)** | **Source** | **Identifier** |
| --- | --- | --- |
| Live/dead cell stain | Invitrogen | L10119 |
| Foxp3/ Transcription Factor Staining Buffer Set | Invitrogen | 00-5523-00 |
| Compensation beads | Invitrogen | 01-2222-42 |
| DNA/RNA extraction kit | Zymo Research | 11309, R1055 |
| MojoSort Mouse CD8 T Cell Isolation Kit | BioLegend | 480007 |

| **Oligonucleotides (5’-3’)** | **Source** | **Identifier** |
| --- | --- | --- |
| PD-L1 ChIP Primer (Human)  Forward GAAGGTCAGGAAAGTCCAAC Reverse TCGGGAAGCTGCGCAGAACT | Reference #25 | N/A |

| PD-L1 ChIP Primer (Mouse)  Forward CGAGCTTCAACCAATCAGCG Reverse GAATTTGCGGTTCTGTCCCG | Reference #25 | N/A |
| --- | --- | --- |
| NR4A1 (Mouse)  Forward ATGCCTCCCCTACCAATCTTC Reverse CACCAGTTCCTGGAACTTGGA | IDT | 1 |
| TOX (Mouse)  Forward TGCCTGGACCCCTACTATTG Reverse CTGGCTGGCACATAGTCCTG | IDT | 2 |
| TOX2 (Mouse) Forward  AGCAGAAACAGGCGTATAAGAGG Reverse GTACATGGGCTGCTTGGGTG | IDT | 2 |
| TBx21 (T-bet) (Mouse)  Forward CAACAACCCCTTTGCCAAAG Reverse TCCCCCAAGCATTGACAGT | IDT | 3 |
| NFAT1 (Mouse)  Forward GTGCAGCTCCACGGCTACAT Reverse GCGGCTTAAGGATCCTCTCA | IDT | 4 |
| IFN-γ (Mouse)  Forward GGATGCATTCATGAGTATTGC Reverse GTGGACCACTCGGATGAG | IDT | 5 |
| Granzyme-B (Mouse)  Forward CCACTCTCGACCCTACATGG Reverse GGCCCCCAAAGTGACATTTATT | IDT | 5 |
| Perforin (Mouse)  Forward GAGAAGACCTATCAGGACCA Reverse AGCCTGTGGTAAGCATG | IDT | 6 |
| GAPDH (Mouse)  Forward AGGTCGGTGTGAACGGATTTG Reverse GGGGTCGTTGATGGCAACA | IDT | 7 |
| NR4A1 KO Exon-2 (Mouse)  Forward CCACGTCTTCTTCCTCATCC Reverse GGCTGGAAGTTGGGTGTAGA | IDT | N/A |
| NR4A1 KO Exon-3 (Mouse)  Forward ACAGAGGGCGCTTTTGTCT Reverse CTCTTGTCCACAGGGCAATC | IDT | N/A |
| NR4A1 KO Exon-4 (Mouse)  Forward CGGACAGACAGCCTAAAAGG Reverse GCAAGACCTCACCTTGGAAT | IDT | N/A |
| siSP1 (Human) | Sigma Aldrich | SASI_Hs02_00333289 (1)  SASI_Hs02_00070994 (2) |
| siSP1 (Mouse) | Sigma Aldrich | SASI_Mm01_00145222 (1)  SASI_Mm01_00145223 (2) |
| siNR4A1 (Human) | Sigma Aldrich | CAGUGGCUCUGACUACUAU (1)  GAGAGCUAUUCCAUGCCUA (2) |
| siNR4A1 (Mouse) | Sigma Aldrich | SASI_Mm01_00077215 (1)  SASI_Mm01_00077216 (2) |
| PD-L1 (Human) | Sigma Aldrich | GGUCAACGCCACAGCGAAUUU (1)  CCUACUGGCAUUUGCUGAACGCAUU (2) |
| PD-L1 (Mouse) | Sigma Aldrich | SASI_Mm01_00062675 (1)  CCCACAUAAAAAACAGUUGTT (2) |
| Scrambled siRNA | Sigma Aldrich | CGU ACG CGG AAU ACU UCG A |

**Supplemental Table S2.** Summary of growth inhibitory IC50 values for CDIM compounds (Supplementary Figures S1)^a^

| **IC50 [µM]** | | | | |
| --- | --- | --- | --- | --- |
| **S.No** | **Compound** | **SW480** | **RKO** | **MC-38** |
| 1 | DIM-3-Br-OCH3 | >10 | >10 | >10 |
| 2 | DIM-3-Cl-5-OCH3 | >10 | >10 | >10 |
| 3 | DIM-3-Br-5-CF3 | >10 | >10 | >10 |
| 4 | DIM-3-Br-5-OCF3 | 9.8 | 7.4 | 7.5 |
| 5 | DIM-3-Cl-5-CF3 | >10 | >10 | >10 |
| 6 | DIM-3,5-Br2 | >10 | 7.5 | 8.4 |
| 7 | DIM-3,5-Cl2 | 10 | 7.2 | 7.2 |

**^a^** The most potent compounds are DIM-3-Br-5-OCF3 (KD = 2.0 µM)(33) and DIM-3,5-Cl2 (KD = 7.7 µM).
